# Survey of psychiatrist use of digital technology in clinical practice

**DOI:** 10.1186/s40345-020-00194-1

**Published:** 2020-10-03

**Authors:** Rita Bauer, Tasha Glenn, Scott Monteith, Peter C. Whybrow, Michael Bauer

**Affiliations:** 1 Department of Psychiatry and Psychotherapy, University Hospital Carl Gustav Carus Medical Faculty, Technische Universität Dresden, Dresden, Germany; 2 ChronoRecord Association, Fullerton, CA USA; 3 Michigan State University College of Human Medicine, Traverse City Campus, Traverse City, MI USA; 4 Department of Psychiatry and Biobehavioral Sciences, Semel Institute for Neuroscience and Human Behavior, University of California Los Angeles (UCLA), Los Angeles, CA USA

## Abstract

**Background:**

Psychiatrists were surveyed to obtain an overview of how they currently use technology in clinical practice, with a focus on psychiatrists who treat patients with bipolar disorder.

**Methods:**

Data were obtained using an online-only survey containing 46 questions, completed by a convenience sample of 209 psychiatrists in 19 countries. Descriptive statistics, and analyses of linear associations and to remove country heterogeneity were calculated.

**Results:**

Virtually all psychiatrists seek information online with many benefits, but some experience information overload. 75.2% of psychiatrists use an EMR/EHR at work, and 64.6% communicate with patients using a new technology, primarily email (48.8%). 66.0% do not ask patients if they use the Internet in relation to bipolar disorder. 67.3% of psychiatrists feel it is too early to tell if patient online information seeking about bipolar disorder is improving the quality of care. 66.3% of psychiatrists think technology-based treatments will improve the quality of care for some or many patients. However, 60.0% of psychiatrists do not recommend technology-based treatments to patients, and those who recommend select a variety of treatments. Psychiatrists use technology more frequently when the patients live in urban rather than rural or suburban areas. Only 23.9% of psychiatrists have any formal training in technology.

**Conclusions:**

Digital technology is routinely used by psychiatrists in clinical practice. There is near unanimous agreement about the benefits of psychiatrist online information-seeking, but research on information overload is needed. There is less agreement about the appropriate use of other clinical technologies, especially those involving patients. It is too early to tell if technology-based treatments or patient Internet activities will improve the quality of care. The digital divide remains between use of technology for psychiatrists with patients living in urban and rural or suburban areas. Psychiatrists need more formal training in technology to understand risks, benefits and limitations of clinical products.

## Background

Digital technology is viewed as fundamental to achieving better care, better patient experience and lower costs (Berwick et al. 2008). Digital technology is also described as having the potential for transformative change in psychiatry in both service delivery and treatments (Bhugra et al. 2017). With the increasing emphasis and investment in technology for all aspects of clinical psychiatry, how psychiatrists perceive technology is critically important.

The objective of this study was to survey psychiatrists to obtain an overview of how they are currently using technology in clinical practice. The survey focused on psychiatrists that treat patients with bipolar disorder, as patients with bipolar disorder use the Internet with the same frequency as the general public, and over 3/4 seek information on bipolar disorder online (Bauer et al. 2016; Conell et al. 2016). Additionally, new technology tools are often thought to be well suited for collecting and analyzing patient data from patients with bipolar disorder (Bauer et al. 2020; Monteith et al. 2016). This survey collected information on the types of products and communications routinely used in clinical practice, the products and websites routinely recommended to patients with bipolar disorder, the technical background of psychiatrists, and general attitudes towards the use of technology. As technology continues to rapidly change, and new products are regularly introduced that modify the practice of psychiatry, more understanding of the psychiatrist perspective is needed.

## Methodology

### Survey

An Internet based survey was created using LimeSurvey software Version 2.56.1 (LimeSurvey 2019), which could be completed from a Windows, Mac, or smartphone device using a web browser. The survey questions focused on 6 areas in addition to demographics: (1) physician communication with patients, (2) patient information seeking about bipolar disorder, (3) technology recommendations for patients, (4) physician information seeking, (5) technology use at work and (6) technology knowledge and attitudes. A small pilot study was completed in Dresden, and the survey questions were refined based on the feedback and results. The survey contained 46 questions and took on average 26.9 min to complete. The survey, and all associated correspondence, were in English. The survey questions along with the psychiatrists responses, except for demographics, are provided in Additional file 1.

### Survey recruitment

Invitations to complete the survey were sent by email to a convenience sample of psychiatrists and others who regularly provide care to those with mental illness. The initial contacts were psychiatrists who participate in international groups related to bipolar disorder. Those who responded were then sent an email with a link and a unique access key to the survey. Those who responded but did not complete the survey were sent a reminder email after several weeks. In addition, 36 respondents provided at least 10 additional contacts. A total of 747 invitations were sent. There were no financial incentives for participating. Data were collected between June and November, 2017.

### Statistics

Descriptive statistics were generated for all survey questions. Many categorical survey responses were summarized into two groups for analysis. For questions that required the respondent to choose one of many options, each option was assigned to a group. For questions that allowed the respondent to choose all options that apply, the respondent was assigned to a group if any options in the group were chosen. The Mantel–Haenszel Chi square test was used to assess association between two variables, such as the association of a response on the use of technology with a demographic variable across multiple strata or countries. Heterogeneity across countries was checked using the Breslow‐Day test, and only results without heterogeneity between countries are reported. A p value of less than 0.05 was considered statistically significant. SPSS Version 25 was used to perform the statistical computations. As this was an exploratory survey, no adjustments were made for multiple comparisons.

## Results

Of the 747 invitations sent, there were 281 completed surveys for a 37.6% response rate. Since 220 of the 281 respondents were psychiatrists, surveys from the small mix of other professions were excluded. Surveys from countries with less than 3 respondents were also excluded, leaving 209 completed surveys from psychiatrists in 19 countries for analysis. The demographic characteristics of the 209 psychiatrists are shown in Table 1. The countries of the 209 psychiatrists are shown in Additional file 1. In this sample, very few statistical differences were found that were due to differences between countries. The results listed below apply across all countries.

**Table 1:** Demographics of the psychiatrists (N = 209)

Demographic category	N	%
Year of birth		
Born before 1980	111	53.1
Born 1980 or later	98	46.9
Gender		
Male	128	61.2
Female	81	38.8
Year graduated from medical school		
Graduated before 2005	100	48.3
Graduated 2005 or later	107	51.7
Years practicing psychiatry		
Practicing less than 15 years	102	48.8
Practicing 15 years or more	107	51.2
Type of Practice		
Private practice, clinic or hospital	113	54.1
Academic hospital or primarily research	96	45.9
Type of Area Most Patients are From		
Urban	166	79.4
Suburban or rural	43	20.6
Percent of patients with mood disorder		
More than 50%	132	63.2
50% or less	77	36.8

### Physician communication with patients

The technology the psychiatrists use to routinely communicate with patients is shown in Table 2-Q11. Overall, 64.6% of psychiatrists communicate with patients using a new technology, primarily standard email (48.8%). When their patients live in urban areas, compared to suburban or rural areas, psychiatrists are more likely to communicate by standard email (55% vs 26%, p = 0.004) and by text message (40% vs 16%, p = 0.009). When their patients live in suburban or rural areas, psychiatrists are less likely to communicate using any new technologies (65% vs 28%, p < 0.001). Psychiatrists vary in perspective on the appropriate uses of email or online communication with patients, as shown in Table 2-Q12. Psychiatrists are more likely to think email or online communication is appropriate for medication refills if they work in academic settings (45% vs. 27%, p = 0.022), or their patients live in urban areas (38% vs. 23%, p = 0.034). Psychiatrists are more likely to think that email or online communications is appropriate for routine clinical follow-ups if they have been practicing for at least 15 years (36% vs. 17%, p = 0.011), or their patients live in urban areas (30% vs. 12%, 0.018). Older psychiatrists, born before 1980, are less likely to think email or online communication is appropriate for administrative issues (62% vs. 78%, p = 0.010). When patients have important clinical information, 33.0% of psychiatrists want patients to schedule an office visit, 28.7% to call the office phone, 18.2% call a personal cellphone, 12.0% email, and 6.7% send a text message.

**Table 2:** Psychiatrist responses to selected questions (N = 209)

Question	N	%	Option
Q11	Do you routinely communicate with patients using any of these technologies? (Check all that apply)		
	29	14	Secure email (encrypted)
	102	49	Standard email
	74	35	Text message
	17	8	Social media
	15	7	Patient portal on an EMR/EHR
	74	35	None of the above
Q12	What do you think are appropriate uses of email or online communication with patients? (Check all that apply)		
	131	63	Notify if symptoms worse
	105	50	Notify if new symptoms
	73	35	Medication refills
	55	26	Routine clinical follow-ups
	63	30	New clinical questions
	138	66	Lab test results
	145	69	Administrative (appointments or billing)
	12	6	None of the above
	9	4	Other
Q21	How would you describe the information that patients find online about bipolar disorder? (Check all that apply)		
	71	34	Increased requests for a diagnosis of bipolar disorder
	90	43	Increased requests for specific medications
	96	46	Increased requests for unnecessary medications or tests
	22	11	Leads to delays in patients seeking help
	26	12	None of the above
	27	13	Don’t know
Q22	Do you think that patient online information seeking is leading to any of these consequences? (Check all that apply)		
	106	51	Improved discussions
	56	27	Improved coping with the illness
	31	15	Increased medication adherence
	45	22	Increased patient confidence
	103	49	Patients better able to express concerns
	47	22	Few patients talk about information found online
	14	7	None of the above
	14	7	Don’t know
Q26	Do you routinely recommend any of these types of websites to patients with bipolar disorder? (Check all that apply)		
	85	41	Sites about mental health or bipolar disorder
	18	9	Sites about general medical information
	14	7	Sites about prescription drug information
	45	22	Government mental heath sites
	12	6	Government prescription drug sites
	10	5	Wikipedia
	86	41	Do not recommend websites
	12	6	Other
Q27	What type of technology-based treatments (online, smartphone apps, or stand alone technologies) do you routinely recommend to patients with bipolar disorder? (Check all that apply)		
	8	4	Online psychotherapy
	12	6	Online patient support groups
	23	11	Active patient monitoring
	13	6	Passive patient monitoring
	34	16	Sleep monitoring
	35	17	Medication adherence support
	48	23	Relaxation techniques
	123	59	Do not routinely recommend technology-based treatments
	5	2	Other
Q29	Do you hesitate in recommending technology-based treatments because of any of these reasons? (Check all that apply)		
	28	13	Privacy considerations
	114	55	Quality of information
	18	9	Patient financial problems
	39	19	Impact on patient behavior
	48	23	Concern about online fraud
	17	8	None of the above
	84	40	Do not routinely recommend technology-based treatment
	7	3	Other

### Patient information seeking—bipolar disorder

Of the respondents, 66.0% do not ask patients if they use the Internet in relation to bipolar disorder. About 23.6% of psychiatrists said at least half of patients with bipolar disorder discussed information found online, while 76.4% said less than half. When psychiatrists ask patients if they use the Internet in relation to bipolar disorder, it is more likely that at least half of patients discuss online findings (39% vs. 15%, p = 0.003). When patients discuss information about bipolar disorder found online with the psychiatrist, 86.6% discuss pharmaceutical treatments and side effects, 57.9% discuss the course of illness and symptoms, 57.9% alternative treatments, and 24.9% experiences at chat rooms and forums. Patients that live in urban areas are more likely to discuss information on the course of illness and symptoms (63% vs 40%, p = 0.021). Patients with bipolar disorder who discuss information found online are viewed as more informed by 64.4% of the psychiatrists, and especially when their patients live in urban areas (69% vs. 47%, p = 0.019). Psychiatrists who view patients that discuss online information as more informed are more likely to ask patients if they use the Internet in relation to bipolar disorder (77% vs. 58%; p = 0.033). The information patients found online about bipolar disorder is viewed as some accurate/some inaccurate by 75.6% of psychiatrists, generating unnecessary fears by 36.4%, relevant by 28.2%, mostly inaccurate by 12.0% and not related to the patient’s diagnosis by 8.1%. When patients discuss information found online, 46.9% of psychiatrists rarely or never review the website, while 53.1% routinely or occasionally review the website. About 42.6% of patients occasionally, and 44.5% rarely request a treatment for bipolar disorder found online. When psychiatrists ask patients if they use the Internet in relation to bipolar disorder, more patients request a treatment for bipolar disorder found online (63% vs 38%, p = 0.003).

Of the psychiatrists, 86.6% feel there are positive consequences to patients searching online for information on bipolar disorder as shown in Table 2-Q22. Other consequences are shown in Table 2-Q21. Those working in academic settings are more likely to have increased patient requests for a diagnosis of bipolar disorder (41% vs. 26%, p = 0.013). Those with patients living in urban areas are more likely to have increased patient requests for unnecessary medications or tests (54% vs 32%, p = 0.048), and more likely see improved coping with the illness (30% vs. 14%, p = 0.040). Of the psychiatrists, 21.2% feel that online information seeking is improving the quality of care, while 67.3% feel it is too early to tell. About half feel it is improving the doctor-patient relationship for some patients and 10.6% for many patients.

### Technology recommendations for patients

Of the psychiatrists, 56.8% routinely recommend web sites to patients with bipolar disorder, with the types of web sites shown in Table 2-Q26. Many patients do not ask for a recommendation, with only 23.4% of respondents saying that more than half of patients ask for a recommendation. More psychiatrists practicing for at least 15 years said that at least half of the patients asked for a recommendation, than those in practice less than 15 years (30% vs. 17%, p = 0.006). Of the psychiatrists that recommend web sites, 40.7% recommend sites about bipolar disorder, and 26.4% recommend government mental health sites.

The technology-based treatments that psychiatrists routinely recommend are shown in Table 2-Q27, although 60.0% of psychiatrists do not routinely recommend these. About half of the psychiatrists would consider the patient’s technical competence before recommending technology-based treatments. Other reasons why psychiatrists hesitate to recommend technology-based treatments are shown in Table 2-Q29. Psychiatrists who do not communicate with patients using any new technologies such as email are less likely to recommend any technology-based treatments (22% vs. 55%, p = 0.010). Of the psychiatrists, 66.3% think technology-based treatments will improve the quality of care for some or many patients, while 33.7% think it will rarely improve quality or do not know. Psychiatrists who routinely ask patients if they use the Internet in relation to bipolar disorder are more likely to believe that technology treatments will improve the quality of care (80% vs. 59%, p = 0.011). Only 23.9% of the psychiatrists provide information to patients on Internet privacy and security. Psychiatrists are more likely to provide information on Internet privacy and security when more than half of their patients have a mood disorder (32% vs. 10%; p = 0.002), and when they rate their technical competency as expert (44% vs. 18%, p = 0.011).

### Physician information seeking/value

Virtually all psychiatrists (98.6%) seek information on the Internet related to bipolar disorder: 72.7% seek information on drugs, 78.9% on drug interactions, 80.4% journal articles, 67.9% practice guidelines, 70.8% evidence based medicine reviews, and 24.4% information to tell patients. Males are more likely to seek journal articles online than females (89% vs. 67%; p = 0.004). Almost all (95%) find access to the vast online information is beneficial, with primary benefits including improved clinical decision making (70.8%), increased confidence in decision making (58.9%), improved patient safety (56.5%), and improved diagnosis (33.5%). Psychiatrists with patients living in urban areas are more likely to find online access leads to improved diagnosis than those with patients living in rural areas (39% vs 14%, p = 0.005). Although 61.8% of psychiatrists find few or no negative consequences of access to so much information, 38.2% of psychiatrists find some negative consequences. These include errors from too much information (21.5%), loss of focus from key issues (20.6%), and a waste of time searching (13.4%).

### Technology at work

In this sample, 75.2% of psychiatrists use an EMR/EHR at work (including partial implementations), while 24.8% do not. More psychiatrists with patients living in urban areas use an EMR compared to those with patients living in suburban or rural areas (78% vs 64%, p = 0.041). Of those who use an EMR, 36.1% use only at work, 21.9% at work and at home, 20.6% at multiple work locations, and 21.3% at multiple work locations and at home. Of the psychiatrists, 66.5% never use telemedicine, 19.4% use 2–3 times per year, 6.8% once a month, and 14.1% more frequently than monthly. Of the psychiatrists, about 66.7% email about patients to other healthcare providers, 42.6% to other physicians in their practice, 29.2% to physicians outside their practice, 8.6% to clinical laboratories, and 5.7% to pharmacies. Of those who email about patients, about half use encrypted email, and half use standard email. For urgent clinical information, 45.4% prefer to be notified by providers using personal cellphone, 28.5% office phone, 10.6% email and 8.7% in-person. For routine clinical information, 46.9% prefer to be notified by email, 15.9% by office phone, 13.0% by personal cellphone, and 15.0% in-person.

### Technical knowledge/attitudes

Virtually all psychiatrists (98.5%) use technology devices at work including a desktop computer (74.2%), smartphone (60.3%), laptop (56.9%), and tablet (20.1%). Males are more likely to use a laptop than females (65% vs. 44%, p = 0.012). The majority of psychiatrists (74.8%) rate their overall technical competency as intermediate or lower, with 25.2% rating themselves as expert. Psychiatrists are more likely to rate themselves as expert when male (32% vs. 15%, p = 0.015), and when they work in academic settings (34% vs. 17%, p = 0.001). Only 23.9% of the respondents have had any formal training in computer science or information technology, unrelated to any demographic. Psychiatrists in practice for less than 15 years are more likely to say it is easy to learn new technology than those in practice for 15 or more years (90% vs. 82%, p = 0.035). Regarding the use of new technologies at work, most psychiatrists (78.0%) are self-taught. Some psychiatrists also learn new technologies from technology staff at work (24.9%), and from medical co-workers (20.6%). Psychiatrists who were born in 1980 or later are more likely to learn from medical co-workers (28% vs. 14%, p = 0.034), and those working in academic settings are more likely to learn from technology staff at work (30% vs. 20%, p = 0.045).

## Discussion

In this survey, virtually all psychiatrists used technology in routine clinical practice. There is near unanimous agreement about the benefits of access to the vast amount of information available online. Psychiatrists regularly go online to find journal articles, practice guidelines, drug prescribing information, and evidence based medicine reviews, and the benefits include improved clinical decision making, increased confidence in decision making, and improved patient safety. However, 38.2% of psychiatrists also reported negative consequences, including errors from too much information, a loss of focus and distraction from key issues, and wasting time. When information overload occurs, more information becomes a burden even if the new information is potentially useful (Bawden and Robinson 2009). Many clinical technologies can generate a large quantity of information for the physician to process and may contribute to information overload. These include data in EMR, alerts from clinical decision support systems, messages from clinicians and administrators, and data generated from patient smartphone apps and wearables (Sittig et al. 2016; Beasley et al. 2011; Bryant et al. 2014; Murphy et al. 2012; Singh et al. 2013).

In contrast to online information-seeking, there is much less agreement on the appropriate use and value of other technologies, especially when patients are directly involved. In this study, many differences are associated with the digital divide between patients who live in rural and urban areas, and the psychiatrist attitudes toward patient use of technology.

### Digital divide

Psychiatrists are more likely to communicate with patients using new technologies and recommend technology-based treatments, when patients live in urban rather than rural or suburban areas. This is consistent with the digital divide (digital inequalities) that persists between urban and rural areas, even in highly digitalized societies in Europe and the US (Perrin 2019; Warf 2019). Access to fast broadband services from home is often unavailable in rural areas of the US, although access to cellular networks has increased (Perrin 2019; Greenberg-Worisek et al. 2019). However, the digital divide extends beyond general Internet access to include inequalities in access to new mobile and Internet of Things (IOT) devices, Internet skills and literacy, ability to afford the ongoing maintenance costs of devices, software and subscriptions, and recognition of online fraud (van Deursen and van Dijk 2019; Lutz 2019). Patients in rural areas use technology less often to find health information than those in urban areas (Greenberg et al. 2018; Rains 2008).

In this survey, more psychiatrists use an EMR at work when patients live in urban rather than rural areas (78% vs 64%). In prior research, hospitals and ambulatory care practices in rural areas often lag in the implementation of advanced EMR functions compared to urban areas (Adler-Milstein et al. 2017; Kim et al. 2017; Rumball-Smith et al. 2018).

### Psychiatrist attitudes towards patient use of technology

About 2/3 of the psychiatrists in this survey do not ask their patients if they use the Internet to seek information on bipolar disorder. This is consistent with a prior survey of 976 patients with bipolar disorder who use the Internet, where about 2/3 of patients rarely or never discussed information learned online with their doctor (Conell et al. 2016). The psychiatrists in this survey vary in their attitude towards patient use of technology. About 2/3 of psychiatrists feel it is too early to tell if patient online information seeking about bipolar disorder is improving the quality of care. When psychiatrists believe that patients who discuss information found online are more informed, they are more likely to ask patients if they seek information online. If psychiatrists do ask patients about Internet use, it is more likely that at least half will discuss their findings, and that patients will request a treatment found online. Psychiatrists should be ready to respond to patient questions about technology, even if they do not recommend it. Since most psychiatrists in this survey find some inaccuracies in what patients read online about bipolar disorder, they may want to suggest reliable online sources of information (Monteith et al. 2013).

Although 2/3 of the psychiatrists think technology-based treatments will improve the quality of care for some or many patients, 60% of psychiatrists do not routinely recommend technology-based treatments for many reasons including the quality of information. The psychiatrists who routinely ask patients if they use the Internet in relation to bipolar disorder are more likely to believe that technology-based treatments will improve the quality of care. Psychiatrists who do not communicate with patients using any type of new technology are less likely to recommend technology-based treatments. Of those who do recommend technology-based treatments, there is variety in what is recommended.

### Psychiatrist technology training

As shown in this survey, a variety of technology is routinely used in the clinical practice of psychiatry, and new applications are available daily. Yet less than a quarter of the psychiatrists have any formal training in technology, unrelated to demographics. Of technologies at work, 78% of the psychiatrists are self-taught users. There is concern that many psychiatrists may not have the background to understand the complex issues of modern technology.

Psychiatrists need an informed framework to evaluate claims for technology products aimed at physicians and patients. This is increasingly important for several reasons: (1) Many new products are based on technologies that psychiatrists may have no clear understanding of, such as AI. (2) There is an atmosphere of constant tech hype to encourage use of new technology by vendors, venture capitalists, entrepreneurs, technology analysts, and university public relations, which is further amplified by social media and sponsored content appearing as technology news across the web (Funk 2019). (3) The limited regulatory requirements for many technology products may not provide the expected assurance of safety and efficacy (Lee and Kesselheim 2018; Parker et al. 2019). (4) Psychiatrists and patients will be interacting with technologies in diverse ways, often previously unimagined.

Psychiatrists need to understand the risks, benefits, and limitations of technology products (Bauer et al. 2020, 2019; Monteith and Glenn 2019; Monteith et al. 2016), to purchase or recommend products from vendors who provide algorithm transparency (IEEE 2019; ACM 2018), and to understand standard security practices to protect themselves and their patients. Importantly, psychiatrists should understand the limitations of their knowledge and skills, when to ask for assistance, and how to select and hire competent technology and cybersecurity consultants.

### Limitations

There are many limitations to this study. The response rate was relatively low, as often reported for online-only surveys of physicians (Sebo et al. 2017; Weaver et al. 2019, Cunningham et al. 2015). Self-reported surveys on timely topics may be subject to response bias, as psychiatrists who are technology enthusiasts may be more likely to have completed the survey. This population may not be generalizable to other psychiatrists. With many younger psychiatrists completing the survey, the results are not well suited for analysis of age-related impacts. The survey did not investigate the features, usability, or efficacy of any specific product such as an EMR or patient smartphone app (Melnick et al. 2019, Bauer et al. 2020). The many challenges associated with automating clinical psychiatry, such as the impacts on physician workflow, multitasking, automation complacency and bias, data quality and algorithm bias, and patient stigma were not discussed (Bauer et al. 2017, 2019). Many studies cited, such as on the use of EMR, were from general medicine rather than psychiatry.

The surveys were completed and this paper submitted before the 2020 pandemic and subsequent increase in technology use in routine medical care. This rapid expansion exacerbated problems such as the digital divide (Renault 2020; Kim et al. 2020), security risks for patients, physicians and medical centers (FBI 2020; Bergal 2020), and inadequately tested apps (Anderson 2020; Leprince-Ringuet 2020). Corrective steps are complex and vary with the specific technology and the healthcare and legal systems in a country.

## Conclusions

In conclusion, psychiatrists routinely use digital technology in clinical practice. There is near unanimous agreement about the benefits of online information-seeking for psychiatrists. Some psychiatrists also report negative effects due to information overload, which needs further investigation. There is less agreement about the appropriate use of other clinical technologies, especially those involving patients. Most psychiatrists think it is too early to tell if patient Internet activities will improve the quality of care, and that technology-based treatments may improve the quality of care for some patients. However, most psychiatrists do not currently recommend technology-based treatments. The digital divide remains between use of technology for psychiatrists with patients living in urban and rural areas. Psychiatrists should be prepared to answer patient questions related to technology. Psychiatrists need more technology training to understand the risks, benefits, and limitations of technology products.

## Supplementary information

**Additional file 1.** MD survey.

**Additional file 2.** Collaborators.

## Data Availability

The data will not be shared or made publicly available.
