# Supplementary material for: Survey of psychiatrist use of digital technology in clinical practice
Source: Int J Bipolar Disord. 2020 Oct 3;8:29. doi: 10.1186/s40345-020-00194-1 (PMC7532734; doi:10.1186/s40345-020-00194-1)
Supplement: Supplementary file 1 — Additional file 1. MD survey. [file 40345_2020_194_MOESM1_ESM.docx]

Survey of psychiatrist use of digital technology in clinical practice

The survey was completed by 209 psychiatrists. For each survey section, the question, instructions to respond, and count of the responses to each possible answer are shown. For example, for question 11, 29 psychiatrists marked that they communicate with patient by secure email (encrypted), 102 by standard email, etc.

Section 1: Physician Communication With Patients

Q11. Do you routinely communicate with patients using any of these technologies? (Check all that apply)

29 : Secure email (encrypted)

102 : Standard email

74 : Text message

17 : Social media

15 : Patient portal on an EMR/EHR

74 : None of the above

Q12. What do you think are appropriate uses of email or online communication with patients? (Check all that apply)

131 : Notify if symptoms worse

105 : Notify if new symptoms

73 : Medication refills

55 : Routine clinical follow-ups

63 : New clinical questions

138 : Lab test results

145 : Administrative (appointments or billing)

12 : None of the above

9 : Other

Q13. How do you prefer patients to contact you about important clinical information? (Check one)

25 : Email

14 : Text message

38 : Personal cellphone

60 : Office phone

69 : Schedule visit

0 : Fax

3 : Other

Section 2. Patient Information Seeking - Bipolar Disorder

Q14. Do you routinely ask patients if they use the Internet in relation to bipolar disorder? (Check one)

71 : Yes

138 : No

Q15. About what percent of your patients with bipolar disorder discuss information with you that they found online about their condition? (Check one)

16 : > 75%

33 : 51-75%

62 : 25-50%

65 : < 25%

32 : < 5%

Q16. What type of information found online do patients frequently discuss in relation to bipolar disorder? (Check all that apply)

121 : Course of illness and symptoms

181 : Pharmaceutical treatments and side effects

12 : Coping skills

24 : Results from symptom checkers/screening tools

52 : Experience at peer support groups, chats and forums

10 : Experience with online therapy

121 : Alternative treatments

11 : Clinical trials

6 : Physician ratings

10 : Online pharmacies

46 : Few patients talk about information found online

3 : None of the above

1 : Other

Q17. How informed are the patients who discuss information found online about bipolar disorder when compared to patients who do not discuss online information? (Check one)

126 : More informed

8 : Less informed

34 : No difference

40 : Few patients talk about information found online

Q18. How would you describe the information that patients find online about bipolar disorder? (Check all that apply)

59 : Relevant for the patient

13 : Too complicated to discuss

13 : Mostly accurate

25 : Mostly inaccurate

158 : Some accurate/some inaccurate

17 : Not related to patient's diagnosis

76 : Generates unnecessary fears

10 : Time consuming to discuss

5 : None of the above

Q19. When patients discuss information found online, do you review the website to verify? (Check one)

37 : Routinely

74 : Occasionally

65 : Rarely

33 : Never

Q20. About how often do patients request a treatment for bipolar disorder that they found online? (Check one)

8 : Routinely

89 : Occasionally

93 : Rarely

19 : Never

Q21. How would you describe the information that patients find online about bipolar disorder? (Check all that apply)

71 : Increased requests for a diagnosis of bipolar disorder

90 : Increased requests for specific medications

96 : Increased requests for unnecessary medications or tests

22 : Leads to delays in patients seeking help

26 : None of the above

27 : Don't know

Q22. Do you think that patient online information seeking is leading to any of these consequences? (Check all that apply)

106 : Improved discussions

56 : Improved coping with the illness

31 : Increased medication adherence

45 : Increased patient confidence

103 : Patients better able to express concerns

47 : Few patients talk about information found online

14 : None of the above

14 : Don't know

Q23. Is patient online information seeking about bipolar disorder improving the quality of care? (Check one)

44 : Yes

21 : No

140 : Too early to tell

3 : None of the above

Q24. Is patient online information seeking improving the doctor-patient relationship for patients with bipolar disorder? (Check one)

22 : For many patients

106 : For some patients

23 : Rarely

18 : No

26 : Few patients talk about information found online

13 : Don't know

Section 3. Technology Recommendations For Patients

Q25. About what percent of patients ask you to recommend websites about bipolar disorder? (Check one)

2 : > 75%

14 : 51-75%

33 : 25-50%

63 : < 25%

97 : < 5%

Q26. Do you routinely recommend any of these types of websites to patients with bipolar disorder? (Check all that apply)

85 : Sites about mental health or bipolar disorder

18 : Sites about general medical information

14 : Sites about prescription drug information

45 : Government mental heath sites

12 : Government prescription drug sites

10 : Wikipedia

86 : Do not recommend websites

12 : Other

Q27. What type of technology-based treatments (online, smartphone apps, or stand alone technologies) do you routinely recommend to patients with bipolar disorder? (Check all that apply)

8 : Online psychotherapy

12 : Online patient support groups

23 : Active patient monitoring

13 : Passive patient monitoring

34 : Sleep monitoring

35 : Medication adherence support

48 : Relaxation techniques

123 : Do not routinely recommend technology-based treatments

5 : Other

Q28. Do you consider the patient's level of technical competence before recommending technology-based treatments? (Check one)

105 : Yes

6 : No

6 : Cannot judge technical competence

92 : Do not routinely recommend technology-based treatments

Q29. Do you hesitate in recommending technology-based treatments because of any of these reasons? (Check all that apply)

28 : Privacy considerations

114 : Quality of information

18 : Patient financial problems

39 : Impact on patient behavior

48 : Concern about online fraud

17 : None of the above

84 : Do not routinely recommend technology-based treatment

7 : Other

Q30. Do you routinely provide information about Internet privacy and security to your patients? (Check one)

50 : Yes

159 : No

Q31. Do you think that technology-based treatments for bipolar disorder will improve the quality of care? (Check one)

43 : For many patients

95 : For some patients

7 : Rarely

44 : Too early to tell

3 : None of the above

16 : Don't know

Section 4. Physician Information Seeking/Value

Q32. What type of information do you regularly seek on the Internet related to patients with bipolar disorder? (Check all that apply)

152 : Drug information

165 : Drug-drug interactions

22 : Help with diagnosis

28 : Specific psychotherapies

168 : Journal articles

142 : Practice guidelines

148 : Evidence based medicine reviews

51 : Information to tell patients

23 : General background

3 : None regularly

Q33. What are the primary benefits to you of having easy access to the vast amount of information online about bipolar disorder? (Check all that apply)

70 : Improved diagnosis

148 : Improved clinical decision making

118 : Improved patient safety

29 : More time available for patient contact

123 : Increased confidence in decision making

27 : Less stress

10 : Few actual benefits

3 : None of the above

Q34. Do you find that having easy access to the vast amount of online information available on bipolar disorder leads to any negative consequences? (Check all that apply)

28 : Substantial waste of time looking for information

43 : Loss of focus, or distraction from key issues

11 : Delays in decision making

45 : Errors from too much information

22 : Less time available for patient contact

12 : Increased stress

76 : Few negative consequences

53 : None of the above

Section 5. Technology At Work

Q35. Do you use an EMR/EHR at work? (Check one)

119 : Yes

36 : Yes, but partially implemented

51 : No

Q36. From what work locations do you use the EMR/EHR? (Check one)

56 : Primary work location only

34 : Primary work location and at home

32 : From all work locations

33 : From all work locations and at home

Q37. Do you participate in telemedicine visits (remote visits via technology)? (Check one)

9 : More than once a week

6 : About once a week

14 : About once a month

40 : About 2-3 times per year

137 : Never use

Q38. Do you communicate by email about patients to any of the following? (Check all that apply)

99 : Staff

89 : Physicians in your practice

61 : Physicians outside your practice

12 : Pharmacies

18 : Clinical laboratories

68 : Do not email about patients

Q39. If you email about patients to other providers which of the following do you routinely use? (Check one)

68 : Secure email (encrypted)

69 : Standard email

68 : Do not email about patients

Q40. How do you prefer physicians and staff to contact you about urgent/emergent clinical information? (Check one)

22 : Email

14 : Text message

94 : Personal cellphone

59 : Office phone

18 : In-person meeting

0 : Fax

0 : Other

Q41. How do you prefer physicians and staff to contact you about routine clinical information? (Check one)

97 : Email

12 : Text message

27 : Personal cellphone

33 : Office phone

31 : In-person meeting

3 : Fax

4 : Other

Section 6. Technical Knowledge/Attitudes

Q42. What devices do you routinely use for work? (Check all that apply)

155 : Desktop computer

119 : Laptop computer

42 : Tablet

126 : Smartphone

3 : None

1 : Other

Q43. How would you rate your overall technical competency? (Check one)

25 : Basic

129 : Intermediate

52 : Expert

0 : Rarely use technology for work

0 : Other

Q44. How do you usually learn new technologies used at work? (Check all that apply)

163 : Self-taught

15 : Vendor provided training

52 : Training provided by technology staff at work

19 : Training provided by technology consultants

43 : Training provided by medical co-workers

24 : External training courses

46 : Rely on online help and user guides

1 : Other

Q45. Is it easy for you to learn to use new technologies? (Check one)

177 : Yes

29 : No

0 : Not interested in learning new technologies

Q46. Have you had any formal training in computer science or information technology? (Check one)

48 : Yes

153 : No

Table 1. Primary Work Location by Country

| Country | N |
| --- | --- |
| Australia | 4 |
| Brazil | 7 |
| Canada | 3 |
| Chile | 13 |
| Estonia | 6 |
| Germany | 22 |
| Hong Kong | 6 |
| India | 8 |
| Malaysia | 5 |
| Mexico | 37 |
| Norway | 3 |
| Poland | 13 |
| South Africa | 3 |
| Spain | 13 |
| Turkey | 5 |
| United Kingdom | 3 |
| United States | 13 |
| Total | 209 |
