# Supplementary material for: Survey of psychiatrist use of digital technology in clinical practice
Source: Int J Bipolar Disord. 2020 Oct 3;8:29. doi: 10.1186/s40345-020-00194-1 (PMC7532734; doi:10.1186/s40345-020-00194-1)
Supplement: Supplementary file 2 — Additional file 2. Collaborators. [file 40345_2020_194_MOESM2_ESM.docx]

**Survey of psychiatrist use of digital technology in clinical practice**

**Full list of the DTP Working Group members**

Bernhard Baune^1^, Claudia Becerra^2^, Sule Bicakci ^3^, André Carvalho^4^, Eric Cheung^5^, Alessandro Cuomo^6^, Rodrigo Dias^7^, Andrea Fagolini^8^, Brent P. Forester^9^, Ana Gonzalez Pinto^10^, Diego Hidalgo-Mazzei^11^, Odeta Jankuviene^12^, Sven Janno^13^, Beny Lafer^14^, Erik Roj Larsen^15,16^, Rasmus Licht^17,18^, Mirko Manchia^19,20,21^, Fatima Meza Urzua^2^, Erin Michalak^22^, Blazej Misiak^23^, René Ernst Nielsen^17^, Abigail Ortiz-Dominguez^24^, Yolanda Pica Ruiz^25^, Patryk Piotrowski^26^, Francisco D.R. da Ponte^27^, Danilo Quiroz^28^, MS Reddy^29^, Bharathram Sathur Raghurman^30^, Dan J. Stein^31^, Ahmad Hatim Sulaiman^32^, Leonardo Tondo^33,34,35^, Eduard Vieta^11^, Maj Vinberg^36^, Biju Viswanath^37^, Maria Yoldi^2^, Allan Young^38,39^

^1^Discipline of Psychiatry, School of Medicine, University of Adelaide, Adelaide, SA, Australia

^2^National Institute of Psychiatry Ramón de la Fuente, Mexico City; Mexico

^3^Hacettepe University Faculty of Medicine Department of Psychiatry, Ankara, Turkey

^4^Centre for Addiction and Mental Health, Mood and Anxiety Disorders Program, University of Toronto, Canada

^5^Department of General Adult Psychiatry, Castle Peak Hospital, Hong Kong

^6^Department of Molecular Medicine and Department of Mental Health (DAI) and University of Siena and University of Siena Medical Center (AOUS), Siena, Italy

^7^Bipolar Disorder Research Program, Department of Psychiatry, University of São Paulo Medical School, São Paulo, Brazil

^8^Department of Molecular Medicine and Department of Mental Health (DAI) and University of Siena and University of Siena Medical Center (AOUS), Siena, Italy

^9^McLean Hospital, Division of Geriatric Psychiatry, Harvard Medical School, Belmont, MA, USA

^10^Department of Psychiatry, University Hospital of Alava, University of the Basque Country, CIBERSAM, Vitoria, Spain

^11^Bipolar Disorders Program, Hospital Clinic, University of Barcelona, IDIBAPS, CIBERSAM, Barcelona, Catalonia, Spain

^12^Department of Psychiatry, Kaunas Medical University Hospital, Kaunas, Lithuania

^13^Department of Psychiatry, University of Tartu, Tartu, Estonia

^14^Bipolar Disorder Research Program, Department of Psychiatry, University of São Paulo Medical School, São Paulo, Brazil

^15^Institute of Clinical Research, Research Unit of Psychiatry, University of Southern Denmark, Odense, Denmark

^16^Department of Psychiatry, Psychiatry in the Region of Southern Denmark, Odense, Denmark

^17^Aalborg University Hospital, Psychiatry, Aalborg, Denmark

^18^Department of Clinical Medicine, Aalborg University, Aalborg, Denmark

^19^Section of Psychiatry, Department of Medical Sciences and Public Health, University of Cagliari, Cagliari, Italy

^20^Unit of Clinical Psychiatry, University Hospital Agency of Cagliari, Cagliari, Italy

^21^Department of Pharmacology, Dalhousie University, Halifax, NS, Canada

^22^Department of Psychiatry, University of British Columbia, Vancouver, Canada

^23^Department of Genetics, Wroclaw Medical University, Wroclaw, Poland

^24^Mood Disorders Research Unit, The Royal's Institute of Mental Health Research, Ottawa, ON, Canada

^25^Hospital Ángeles del Pedregal, Mexico City, Mexico

^26^Department of Psychiatry, Wroclaw Medical University, Wroclaw, Poland

^27^Department of Psychiatry, Universidade Federal do Rio Grande do Sul, Porto Alegre, Brazil

^28^Department of Psychiatry, Diego Portales University, Santiago de Chile, Chile

^29^Asha Bipolar Clinic, Asha Hospital, Hyderabad, Telangana, India

^30^Department of Psychiatry, National Institute of Mental Health and Neurosciences, India

^31^Department of Psychiatry and Mental Health, South African Medical Research Council Unit on Risk and Resilience in Mental Disorders, University of Cape Town, Cape Town, South Africa

^32^Department of Psychological Medicine, Faculty of Medicine, University of Malaya, Kuala Lumpur, Malaysia

^33^Department of Psychiatry, Harvard Medical School, Boston, MA, USA.

^34^International Consortium for Bipolar & Psychotic Disorders Research, McLean Hospital, Belmont, MA, USA

^35^Lucio Bini Mood Disorder Center, Cagliari, Sardinia, Italy

^36^The Copenhagen Affective Disorder research Centre (CADIC), Psychiatric Centre Copenhagen, Copenhagen, Denmark

^37^Department of Psychiatry, National Institute of Mental Health and Neuro Sciences (NIMHANS), Bangalore, India

^38^Department of Psychological Medicine, Institute of Psychiatry, Psychology and Neuroscience, King's College London, London, UK

^39^South London & Maudsley NHS Foundation Trust, Maudsley Hospital, London, UK
